# Multi-contrast computed tomography atlas of healthy pancreas with dense displacement sampling registration

**DOI:** 10.1117/1.JMI.12.2.024006

**Published:** 2025-04-17

**Authors:** Yinchi Zhou, Ho Hin Lee, Yucheng Tang, Xin Yu, Qi Yang, Michael E. Kim, Lucas W. Remedios, Shunxing Bao, Jeffrey M. Spraggins, Yuankai Huo, Bennett A. Landman

**Affiliations:** a Vanderbilt University, Department of Computer Science, Nashville, Tennessee, United States; b NVIDIA, Santa Clara, California, United States; c Vanderbilt University, Department of Cell and Developmental Biology, Nashville, Tennessee, United States; d Vanderbilt University, Department of Biochemistry, Nashville, Tennessee, United States; e Vanderbilt University, Department of Chemistry, Nashville, Tennessee, United States; f Vanderbilt University Medical Center, Department of Pathology, Microbiology, and Immunology, Nashville, Tennessee, United States; g Vanderbilt University Medical Center, Department of Radiology, Nashville, Tennessee, United States; h Vanderbilt University, Department of Electrical and Computer Engineering, Nashville, Tennessee, United States

**Keywords:** computed tomography, registration, pancreas

## Abstract

**Purpose:**

Diverse population demographics can lead to substantial variation in the human anatomy. Therefore, standard anatomical atlases are needed for interpreting organ-specific analyses. Among abdominal organs, the pancreas exhibits notable variability in volumetric morphology, shape, and appearance, complicating the generalization of population-wide features. Understanding the common features of a healthy pancreas is crucial for identifying biomarkers and diagnosing pancreatic diseases.

**Approach:**

We propose a high-resolution CT atlas framework optimized for the healthy pancreas. We introduce a deep-learning-based preprocessing technique to extract abdominal ROIs and leverage a hierarchical registration pipeline to align pancreatic anatomy across populations. Briefly, DEEDS affine and non-rigid registration techniques are employed to transfer patient abdominal volumes to a fixed high-resolution atlas template. To generate and evaluate the pancreas atlas, multi-phase contrast CT scans of 443 subjects (aged 15 to 50 years, with no reported history of pancreatic disease) were processed.

**Results:**

The two-stage DEEDS affine and non-rigid registration outperforms other state-of-the-art tools, achieving the highest scores for pancreas label transfer across all phases (non-contrast: 0.497, arterial: 0.505, portal venous: 0.494, delayed: 0.497). External evaluation with 100 portal venous scans and 13 labeled abdominal organs shows a mean Dice score of 0.504. The low variance between the pancreases of registered subjects and the obtained pancreas atlas further illustrates the generalizability of the proposed method.

**Conclusion:**

We introduce a high-resolution pancreas atlas framework to generalize healthy biomarkers across populations with multi-contrast abdominal CT. The atlases and the associated pancreas organ labels are publicly available through the Human Biomolecular Atlas Program (HuBMAP).

## 1 Introduction

**Fig. 1:**
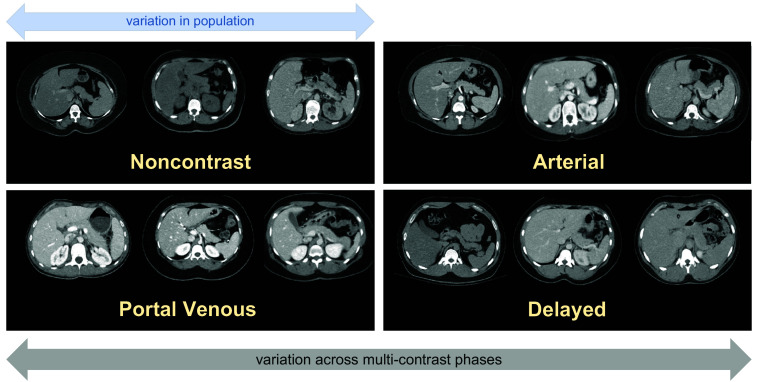
Anatomical characteristics of the pancreas vary widely among the population. Visual differences between each contrast phase are also shown: (a) non-contrast, (b) arterial, (c) portal venous, and (d) delayed. A generalizable reference is needed to adapt individual morphological variability within contrast phases.

With the complex relationship between physiological and metabolic processes in the human body, substantial efforts are underway to map the organization and molecular profiles of cells within specific tissues.^1^ Adapting a multi-scale context from the cellular to the organ level is essential for correlating biomarkers across different imaging domains.^1^ At the system scale, computed tomography (CT) is widely used for visualizing patient anatomy and contextualizing the anatomical and molecular characteristics of specific organs.^2^ In addition, multi-phase contrast-enhanced CT scans provide detailed structural information for improved clinical diagnoses.^3^^–^^5^ During CT imaging procedures, contrast enhancement is performed by injecting a contrast agent prior to imaging. Four different phases are typically defined based on the amount of time that has passed relative to the injection of contrast: (1) non-contrast, (2) arterial, (3) portal venous, and (4) delayed. Imaging at different phases allows better detection of anatomical variations in organs among patients with different demographics (e.g., sex, body size) (Fig. 1).

Among abdominal organs, the pancreas exhibits notable variability in volumetric morphology, shape, and appearance.^6^ The pancreas aids in digestion and releases hormones to regulate blood sugar. Pancreatic cancer has the highest mortality rate of all major cancers.^7^ Therefore, understanding common features of the healthy pancreas is crucial for identifying biomarkers and diagnosing pancreatic diseases. However, these differences in pancreas morphology create difficulties in generalizing population-wide pancreas features. To investigate the population-wise healthy biomarkers of the pancreas at the systemic level, a standardized imaging atlas framework is needed to adapt the population-wise anatomical characteristics onto a single template using image registration techniques, integrating information across scales, and interpreting the results of organ-specific analyses in a common spatial context.

There has been significant development of organ/tissue-specific atlas frameworks for magnetic resonance imaging (MRI), with extensive efforts applied to leveraging MRI to investigate brain biomarkers.^8^^,^^9^ Kovačevi’c et al. proposed a 3D variation atlas of a mouse brain to capture population similarities and variance in anatomy.^10^ Wang et al. created a population average reference framework using 1675 scans of mouse brain MRIs.^8^ Shi et al. created an unbiased infant brain atlas using group-wise registration from three different scanning time points with MRI from 56 males and 39 females.^11^ Kuklisova-Murgasova et al. proposed multiple probabilistic atlases to generalize the aging characteristics of infants aged 29 to 44 weeks.^12^ Gholipour et al. generated an unbiased spatial-temporal 4D atlas and time-variable longitudinal atlas for the infant brain.^13^ To further investigate the aging characteristics in the brain tissue, Zhang et al. used patch-based registration in the spatial-temporal wavelet domain to generate a longitudinal atlas.^14^ Although previous efforts focused on generating healthy brain atlas templates, Rajashekar et al. proposed high-resolution normative atlases to visualize population-wise representations of brain diseases (e.g., brain lesions, stroke) in elderly populations using both FLAIR MRI and non-contrast CT modalities.^15^ For abdominal regions, pioneer studies have developed a multi-contrast kidney atlas that generalizes morphological characteristics within kidney organs.^16^^,^^17^ The kidney atlas template was further extended to organ substructures (e.g., medulla, renal cortex, pelvicalyceal systems) for arterial phase CT.^18^ However, due to the variation in organ anatomies and body sizes across the population, generating a standard reference template for the pancreas remains challenging, with no publicly available pancreas atlas.

In addition to the development of tissue/organ atlases, robust image registration algorithms to map the anatomical context of a patient onto such a template are important. Previous works have sought to enhance registration performance by innovating conventional frameworks with both affine and deformable transformations.^19^^–^^21^ Spatial transformation is optimized by regularizing the deformation field to align the anatomical context from the moving image to a single fixed template using traditional approaches such as discrete optimization,^22^ B-spline deformation,^23^ Demons,^24^ and symmetric normalization.^20^ Xu et al. demonstrated the superiority of dense displacement sampling (DEEDS) in non-rigid registration for abdominal CT scans.^25^ Deep learning approaches have also been explored to accelerate the registration process. VoxelMorph is a deep neural network approach initially developed for brain image registration that optimizes with a generalized function to compute a deformation field in an unsupervised manner.^26^^,^^27^ Zhao et al. extended the adapted VoxelMorph framework as a recursive cascaded network that leveraged organ labels to crop organ-specific regions of interest (ROIs) and progressively registered the anatomical context to the fixed template.^28^ Nonetheless, abdominal image registration requires a larger deformation field due to significant inter-subject variation in body size and organ morphology. Consequently, deep learning-based approaches often fail to maintain the characteristics of abdominal organs and tend to over-smooth the transformation, underperforming traditional optimization tools such as ANTs and NiftyReg when the variations between subjects are high.^29^ In addition, the registration tools above also fail to accurately provide the deformation field when the reference image and moving images show different fields of view due to variations in imaging protocols or human anatomy. Therefore, deep learning-based preprocessing has been integrated with conventional registration methods to enhance registration accuracy. Lee et al. performed body part regression (BPR) prior to registration to minimize field of view (FOV) differences between scans, thus reducing the failure rate of registration.^16^

In this work, we propose a high-resolution CT pancreas atlas framework optimized for a healthy pancreas using DEEDS registration. Initially, we crop the abdominal ROI from each subject scan with a deep neural network BPR to align the FOV between the template and subject scans. After cropping, a two-stage hierarchical registration pipeline is employed to register each subject scan to the high-resolution atlas target with metric-based registration across all contrast phases.^30^^,^^31^ To evaluate the quality of anatomical transfer across scans, we compute average and variance mappings to demonstrate the organ appearance across all registered outputs in each phase and further quantify the registration performance with inverse label transfer from our atlas framework. The pancreas segmentation label of each subject is also registered to the target label, and a final segmentation label on the atlas is obtained through majority voting, enhancing the visualization and localization of the pancreas within the atlas. Overall, our main contributions are summarized as five folds:

• We establish the first standardized framework for creating a population-based 3D pancreas CT atlas.
• We propose the first standardized hierarchical metric-based framework optimized for a healthy pancreas to generalize the anatomical and contrast characteristics of the organ across different demographics and the domain shift due to contrast phases.
• We compare three common registration tools (e.g., ANTs, NiftyReg, DEEDS) for abdominal CT scan registration, demonstrating the superiority of DEEDS for registering all abdominal organs.
• We evaluate the effectiveness of our proposed atlas template by inversely transforming the atlas target labels to a de-identified research cohort of 100 abdominal scans with 13 ground-truth organ labels. Another large cohort of unlabeled multi-contrast phase CT scans is leveraged to compute average and variance mappings, demonstrating the generalizability of the atlas framework. Our proposed atlas framework achieves stable transferability in the pancreas region with an average Dice score of 0.5 across different contrast phases (non-contrast: 0.497, arterial: 0.505, portal venous: 0.494, delayed: 0.497) in an unsupervised setting.
• The average template and the associated pancreas organ labels are publicly available through the Human Biomolecular Atlas Program (HuBMAP).

## 2 Methods

### 2.1 Framework Overview

**Fig. 2:**
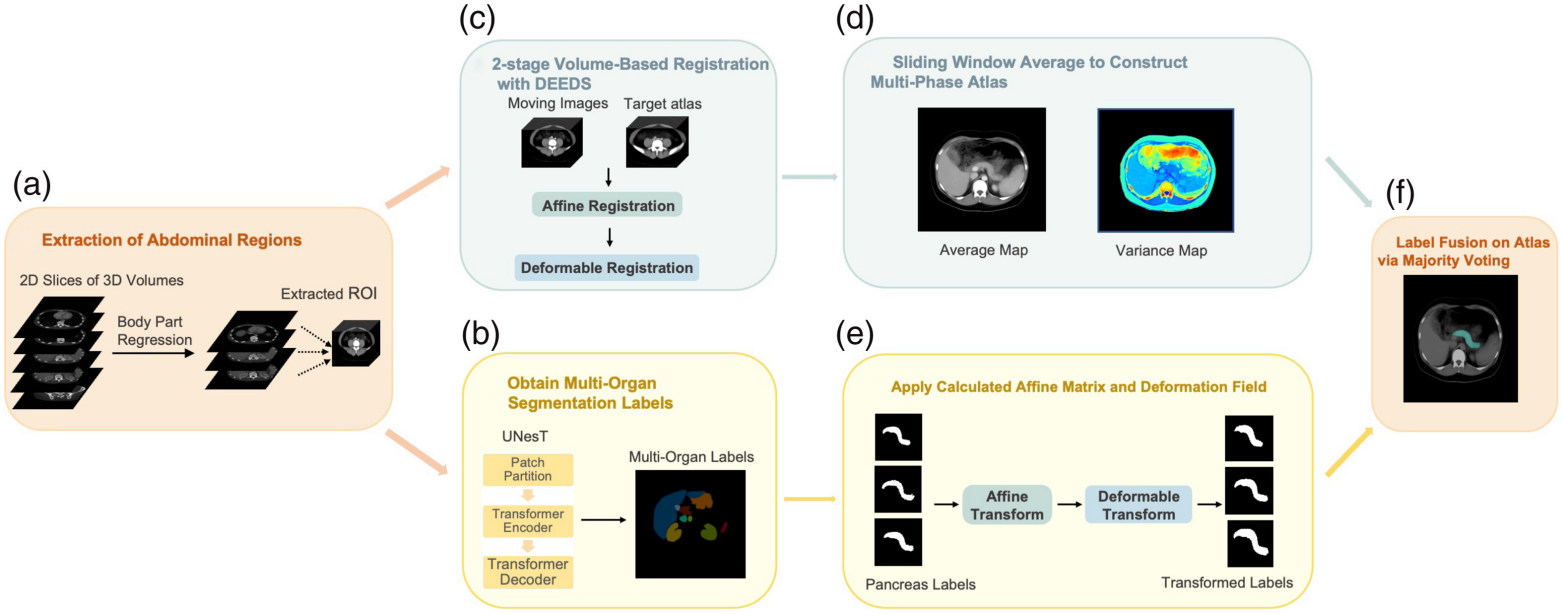
Complete overview of our proposed atlas generation framework. We extract the abdominal regions from CT scans using the body part regression network. We register the cropped ROI to the reference image with a hierarchical two-stage registration and compute both average and variance mappings to evaluate the effectiveness of the atlas framework. Furthermore, we statistically fuse the pseudo-predictions from the transformer-based segmentation network UNesT and perform inverse transformation back to the subject space for evaluation.

The framework for constructing a healthy CT pancreas atlas involves six major steps, as shown in Fig. 2. To acquire the atlas for each contrast phase, we registered subjects from various phases to a high-resolution single-subject template used as the reference image. Initially in step A, we employed a deep neural network BPR to extract the ROI from the original 3D volumes. This step is crucial for aligning the moving subjects with the template subject, thereby reducing registration failure. Subsequently in step B, the volume-based registration tool DEEDS was used to register the cropped scans to the template subject through a two-stage registration involving both affine and deformable transformations. Following registration in step D, we calculated the average map from all registered subjects using the sliding window averaging algorithm and computed the variance map between the atlas and the registered subjects to assess the atlas’s stability. In parallel with the volume registration, as shown in step B, we performed segmentation on the extracted 3D volumes, obtaining labels for 13 abdominal organs using the transformer-based network UNesT. In step E, the obtained pancreas label was further registered to the reference label by applying the affine and deformable fields derived from the DEEDS registration. Finally, in step F, we achieved a statistically fused pancreas label through majority voting on each voxel, which was overlaid with the average map. This process was repeated for each contrast phase, resulting in four distinct pancreas atlases corresponding to the non-contrast, arterial phase, portal venous phase, and delayed phase.

### 2.2 Self-Supervised Body Part Regression Network for Preprocessing

With substantial variation in imaging protocols, imaging samples from a large cohort usually result in different ranges of fields of view (FOVs). Such variability in FOVs may increase the failure rate of registration when the FOV difference between the subject scan and the atlas template is large. Here, we adapt the body part regression (BPR) network (https://github.com/MIC-DKFZ/BodyPartRegression) to extract a similar FOV within the abdominal regions only and to enhance registration performance.^32^ Specifically, given an unlabeled dataset $\{x_{ui}{\}}_{i=1}^{N}$ as the moving image domain and the atlas image $x_{a}$, our goal is to crop the volumetric $x_{ui}$ to have an approximate FOV of abdominal interest only with the atlas template $x_{a}$. Tang et al. proposed a self-supervised BPR network to generate a continuous score for each axial slice in the volumetric scans as the normalized body localization values.^32^^,^^33^ Each score is within the range of −12 to +12, which refers to different anatomical locations (e.g., −12, upper chest; −5, diaphragm/upper liver; 4, lower retroperitoneum; 6, pelvis). For the abdominal region, we limit the score ranges across all axial slices within −6 to 5 and crop the slices accordingly.

### 2.3 Transformer-Based Segmentation Network

**Fig. 3:**
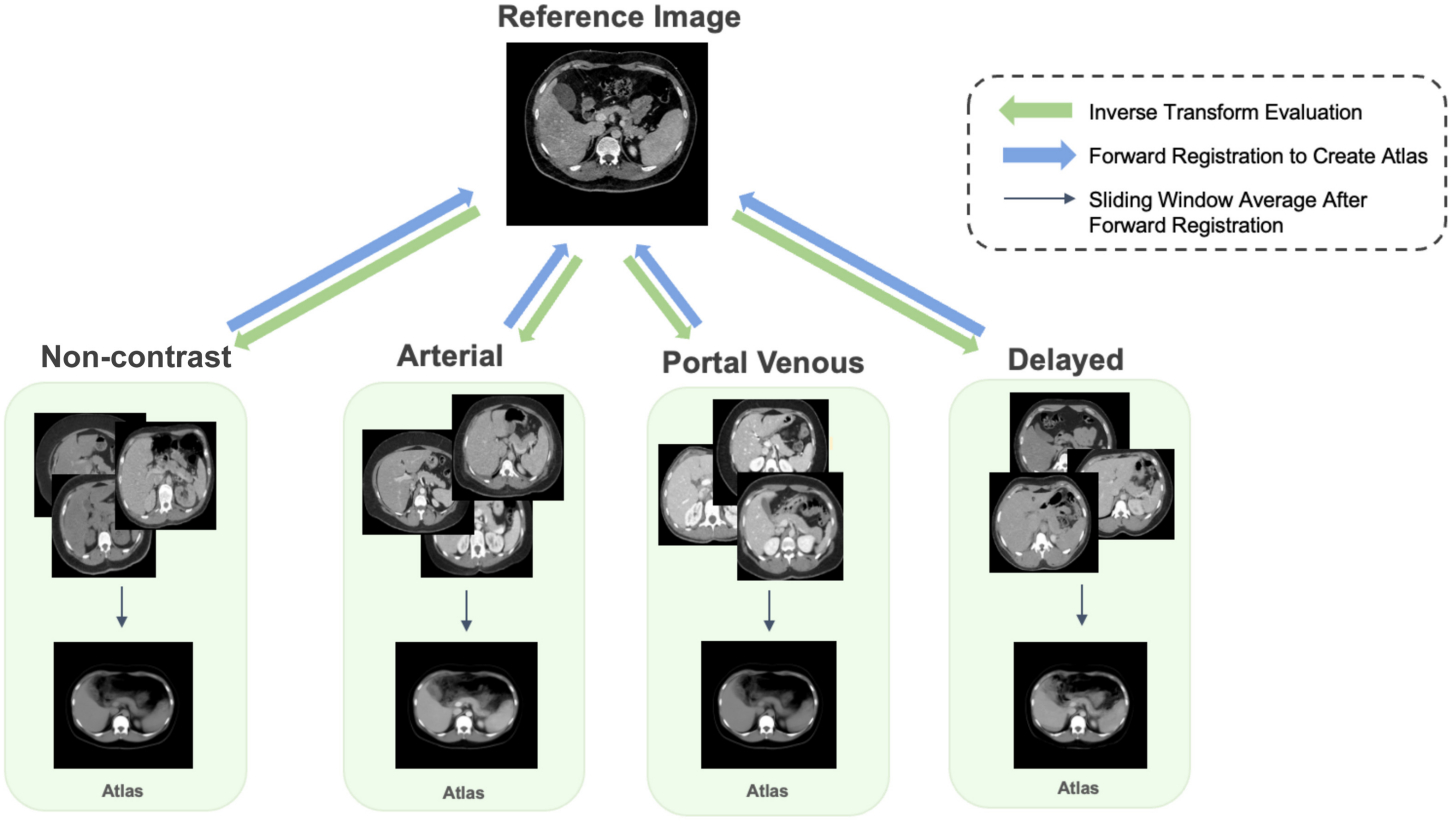
Transformation is bidirectional between the atlas target and the original CT scans. The forward registration transforms CT scans from each contrast phase into the reference image space and performs a sliding window average on the registered subjects, creating the final average atlas. To evaluate the effectiveness of our atlas framework, inverse transformation is performed to backpropagate the anatomical label from the atlas template to the moving subject domain and quantify the similarity with the corresponding ground truth label.

The need for segmentation labels on abdominal scans in our study is twofold: (1) inverse transform evaluation and (2) joint label fusion to visualize the pancreas region on atlases. Pancreas organ labels are essential both to identify healthy biomarkers across populations and to perform statistical label fusion, generating the atlas label that is overlaid on the created 3D abdominal atlas. Multi-organ labels are needed to evaluate the registration performance and the stability of the atlas through inverse transformation, as indicated in Fig. 3. With the recent advance of deep neural networks in medical image segmentation,^34^^–^^36^ transformer-based networks have been proposed for their proficiency in capturing long-range dependencies and having a large receptive field.^37^^,^^38^ Here, we adapt a transformer-based model that incorporates a hierarchical design alongside a block aggregation method, achieving state-of-the-art performance across multiple modalities and public datasets.^39^ This model initially projects 3D volumes into a sequence of patches and then employs a 3D block aggregation algorithm to enhance communication between these patches. Within each hierarchical level, patches are transformed into blocks and introduced to the transformer layer. The output then undergoes the block aggregation algorithm and is subsequently downsampled for the next hierarchy. The model consists of three hierarchies with configurations of 64, 32, and 1 block(s), respectively. The block aggregation algorithm leverages local attention, thereby enhancing data efficiency.

### 2.4 2-Stage Hierarchical Registration

Our registration framework consists of two main steps: (1) affine registration and (2) deformable registration. Inspired by Lee et al.,^17^^,^^18^ we adapt dense displacement sampling (DEEDS) as our registration backbone for abdominal imaging. The idea behind DEEDS is to compute a large deformation field with a discretized sampling space to align the anatomical context across abdominal organs with variable morphology.^30^^,^^31^^,^^40^ First, we perform DEEDS affine registration to align the abdominal organs with 12 degrees of freedom from the moving images to the atlas template. The affine-aligned intermediates are then leveraged for the DEEDS deformable registration as our second-stage registration process. The DEEDS deformable registration refines the spatial relationship between randomly selected patches and computes the local voxel-wise correspondence with its specific similarity metric as follows:

$$D(x_{m},p_{x},p_{y})=\exp(\frac{S(p_{x},p_{y})}{q^{2}}),\;p_{x},p_{y}\in N,$$ (1)

where $p_{x}$ and $p_{y}$ are denoted as the center coordinate of another patch from one of the neighborhood N, S is the self-similarity metric, which is optimized by a distance function D between the image patches from the moving samples. $q^{2}$ denotes a function to evaluate noise from both local and global perspectives. This similarity metric mitigates the adverse effects of image artifacts or random noise from the central extracted patch. During deformable registration, five different levels are utilized in grid spacing, ranging from eight to four voxels to randomly select patches. The displacement search radii are defined from six to two steps between five and one voxel. Six neighborhoods are chosen to compute 12 distances between pairwise patches for optimization.^30^^,^^31^^,^^40^ Both deformed scans and the corresponding displacement matrix are generated as the final output, aligning the fine-grain anatomical details of each organ.

### 2.5 Weighted Sliding Window Average

To reduce the blurring and maintain the characteristics of the organ on the average atlas, the weighted sliding window averaging algorithm on 3D patches is used to compute the average atlas from registered subjects. For i’th patch on k’th registered volume, the weighted template μ is updated iteratively with the formula below:

$$\mu_{k+1}=\frac{\underset{i}{\sum }x_{k,i}w_{k,i}}{\underset{i}{\sum }w_{k,i}},$$ (2)

$$w_{k,i}=\frac{1}{\left\|x_{k,i}-\mu_{k}\right\|}.$$ (3)

## 3 Experimental Setup

### 3.1 Datasets

#### 3.1.1 Clinical research multi-phase CT cohort

The dataset was sourced from the research derivative (RD) database, which includes clinical and related data, and ImageVU, a research repository of medical imaging data and image-related metadata. Multi-contrast phase CT volumes were selected for the formation of multi-phase atlases from a large cohort of 2000 abdominal CT scans, conducted under the approval of the Institutional Review Board (IRB #131461). These scans encompass retroperitoneal and abdominal organs. To construct a healthy CT atlas optimized for the pancreas region, patients exhibiting pancreatic diseases were excluded, resulting in a cohort of 898 subjects aged 18 to 50 years. Quality assessment was conducted on these registered subjects, involving a visual examination and the calculation of the inverse Dice score of the pancreas. Subjects with a pancreas Dice score below 0.1 were excluded. This selection process yielded 443 unlabeled CT volumes used to create the atlas across different contrast phases: 59 volumes for the non-contrast phase, 40 volumes for the arterial phase, 330 volumes for the portal venous phase, and 14 volumes for the delayed phase. All volumetric scans were initially reoriented to the standard RAS (right-anterior-superior) orientation before data preprocessing.^41^ During preprocessing, intensity values outside the body (e.g., CT bed) were set to −1000 Hounsfield units (HU), and the volumes were thresholded using a soft tissue window ranging from −275 to 275 HU. BPR was then applied to isolate the FOV in the abdominal region and align it with the target image’s FOV. Organ segmentation labels for the selected subjects were obtained from the UNesT model. The cropped scans were resampled using bicubic interpolation to match the resolution and dimensions of the atlas template for registration, whereas organ labels were resampled using nearest neighbor interpolation to conform to the atlas dimensions.

#### 3.1.2 Multi-organ labeled portal venous abdominal CT cohort (BTCV)

A separate healthy clinical cohort consisting of 100 de-identified portal venous phase abdominal CT scans was included in this dataset. Of these, 20 volumetric scans were part of the publicly available test set from the MICCAI 2015 Multi-Atlas Abdomen Labeling (BTCV) challenge. All volumetric scans are labeled with 13 organs, including (1) spleen, (2) right kidney, (3) left kidney, (4) gall bladder, (5) esophagus, (6) liver, (7) stomach, (8) aorta, (9) inferior vena cava (IVC), (10) portal splenic vein (PSV), (11) pancreas, (12) right adrenal gland (RAD), and (13) left adrenal gland (LAD). The image size of each scan is 512×512×[80,255] with a spacing of [0.54, 0.98] mm × [0.54, 0.98] mm × [2.5, 7.0] mm. During preprocessing, the intensity values outside of the body (i.e., CT bed) were set to −1000  HU, and the volumes were then thresholded using a soft tissue window (HU range from −275 to 275). BPR was also performed on each scan to isolate the FOV in the abdominal region and match the FOV of the target image. The cropped scans were then resampled using bicubic interpolation to align with the resolution and dimensions of the atlas template for registration. The organ labels were similarly resampled to match the dimensions of the atlas labels using nearest neighbor interpolation. To evaluate the generalizability of our proposed atlas framework and compare the performance of different registration methods, we used all 100 volumes to calculate the inverse Dice score for the 13 organs. In addition, 20 subjects were used in the ablation study to determine the optimal range of BPR scores to define the best FOV correlation between all moving images and the atlas target. We also utilized a pretrained UNesT model, which was trained on 80 subjects from this dataset and evaluated on the 20 testing subjects.^39^

#### 3.1.3 High-resolution single subject atlas template

Inspired by Ref. 17, we selected the atlas template under several conditions: (1) high resolution in both in-plane and through-plane, (2) distinctive contrast appearance in the pancreas organ morphology with clear boundaries, and (3) healthy condition. The atlas template is a portal venous phase CT scan provided by HuBMAP with a high resolution of 0.8×0.8  mm×0.8  mm and corresponding dimensions of 512×512×434. The atlas template is annotated with 13 organs by performing statistical label fusion using the pseudo-segmentations from all 898 registered subjects. The intensity values outside the body (i.e., CT bed) were set to −1000  HU, and the volume was then thresholded using the soft tissue window (HU range from −275 to 275).

### 3.2 Implementation Details

BPR and UNesT are pretrained models obtained from previous studies.^32^^,^^39^ The BPR model is a U-Net-like architecture trained with a total of 230,625 2D slices from multi-center datasets including 1030 whole-body CT scans. The BPR model was optimized end-to-end using the Adam optimizer with a learning rate of 0.0001 and a batch size of 4.^32^ The UNesT model is a nested transformer network trained on 80 subjects from the BTCV dataset, with an input volume size of 96×96×96, an initial learning rate of 1e–4, and a weight decay of 1e–4 for 100 K iterations. The segmentation performance on 20 testing subjects is reported in the paper.^39^ The model achieves high accuracy across all organs, with an average Dice score of 0.843 and a Dice score of 0.806 for the pancreas. We directly implemented the pretrained BPR model to crop the 3D volumes of the multi-phase CT dataset and BTCV data for two-stage registration. The pretrained UNesT model was used to obtain the pseudo-segmentation labels for the unlabeled multi-phase CT data, which were then used to create the fused pancreas label overlaid on the constructed atlases.

To ensure the accuracy of the average atlas created from registered subjects, we investigated the effectiveness of current state-of-the-art registration tools. We used two datasets to compare different registration methods: the unlabeled multi-phase CT clinical scans and the labeled multi-organ portal venous BTCV data. The registration accuracy of the pancreas region on multi-phase CT scans for all phases was evaluated using the inverse label transformation on the pseudo-labels obtained from the UNesT model, whereas the labeled BTCV data were used as an external validation dataset to compare different registration methods across all 13 abdominal organs. To evaluate the registration performance, we performed an inverse label transform from the atlas back to the space of each participant for 13 segmentation labels in abdominal regions on 100 labeled portal venous CT volumes from the BTCV dataset. The two-stage hierarchical registration was performed for all preprocessed CT volumes. We conducted extensive analysis with three registration tools, including ANTS,^20^^,^^25^ NIFTYREG,^25^^,^^42^ and DEEDS^30^^,^^31^^,^^40^ for metric-based registration with our multi-organ labeled portal venous CT cohort. All three methods are initialized with affine registration followed by non-rigid registration. For ANTS, we used ANTS-QUICK-MI, which begins with the alignment of the center of mass, initializes the affine registration using mutual information as the similarity metric, and employs symmetric normalization (SyN) transformation for non-rigid registration, using the maximum convergence iterations of 1000 for affine and 300 for non-rigid registration. NIFTYREG uses five levels of multi-resolution sampling for both affine and non-rigid registrations. The affine registration for NIFTYREG is based on a block-matching approach and trimmed least square optimization with a maximum iteration of 5 per level, whereas the non-rigid registration for NIFTYREG is based on a block-matching approach and free-form deformation, with a maximum convergence iteration of 1000. Similarly, DEEDS uses five scale levels with grid spacings ranging from 8 to 4 voxels, displacement search radii from 6 to 2 steps with quantizations between 5 and 1 voxels. The regularization weighting was set to 0.4. Self-similarity context descriptors^30^ were derived, and their Hamming distances between images were used to guide the local displacement. The non-rigid registration was initialized using an affine registration based on the same similarity metric, a similar block-matching search, and trimmed least squares.

After validation of the registration tools and BPR parameters, the final average atlases for each contrast phase were obtained by applying a weighted sliding window averaging algorithm on 443 registered subjects from the clinical multi-phase CT cohort. With a patch size of 96×96×96 and a patch overlap of 0.75×0.75×0.75. The pseudo-pancreas labels were also deformed into the reference space using the affine matrix and deformation field derived from DEEDS registration. The registered labels were then fused to create the pancreas label overlaid on the average atlas. In addition, the Dice score for the pancreas label after inverse transformation was calculated to demonstrate the reliability of the registration.

### 3.3 Evaluation Metrics

We adapted two commonly used metrics to evaluate the similarity between the inverse transferred labels from atlas space to moving subject space and the corresponding moving ground truth label: (1) Dice similarity score and (2) Hausdorff distance (HD). The definition of Dice score is to compute the overlapping ratio between the predicted pixel/voxel-wise label and the ground truth label. The Dice score is defined as follows:

$$\text{Dice}\;(P,G)=\frac{2|P\cap G|}{\left|P|+|G\right|},$$ (4)

where P denotes as the label prediction and G is the corresponding ground truth label while ∥ denotes as L1 normalization. For computing the Hausdorff distance, we extracted the three-dimensional coordinates of each vertice from the surface rendering of both the predicted label and the ground truth. We computed the Hausdorff distance with the vertices as follows:

$$\mathrm{HD}(v_{p},v_{g})=\underset{v_{p}\in P}{\sup}\;\underset{v_{g}\in G}{\inf}\;\mathrm{Dist}(v_{p},v_{g}),$$ (5)

where $v_{p}$ and $v_{g}$ denotes as the vertex coordinates of the label prediction and ground-truth label respectively. Sup and inf refer to the upper and lower bound respectively for the distance function Dist value, which is the Euclidean distance between two points.

## 4 Results

### 4.1 Evaluation with Clinical Research Cohort and Multi-Organ Labeled Cohort

After cropping the abdominal regions from the raw data using BPR, we resampled the cropped volumes and registered them to the reference volume using DEEDS. We then qualitatively assessed the registered subjects and removed those with registration failures, ending up with 443 subjects in total for four contrast phases. The average atlases for four contrast phases were subsequently obtained by applying a weighted sliding window average on the registered volumes.

**Fig. 4:**
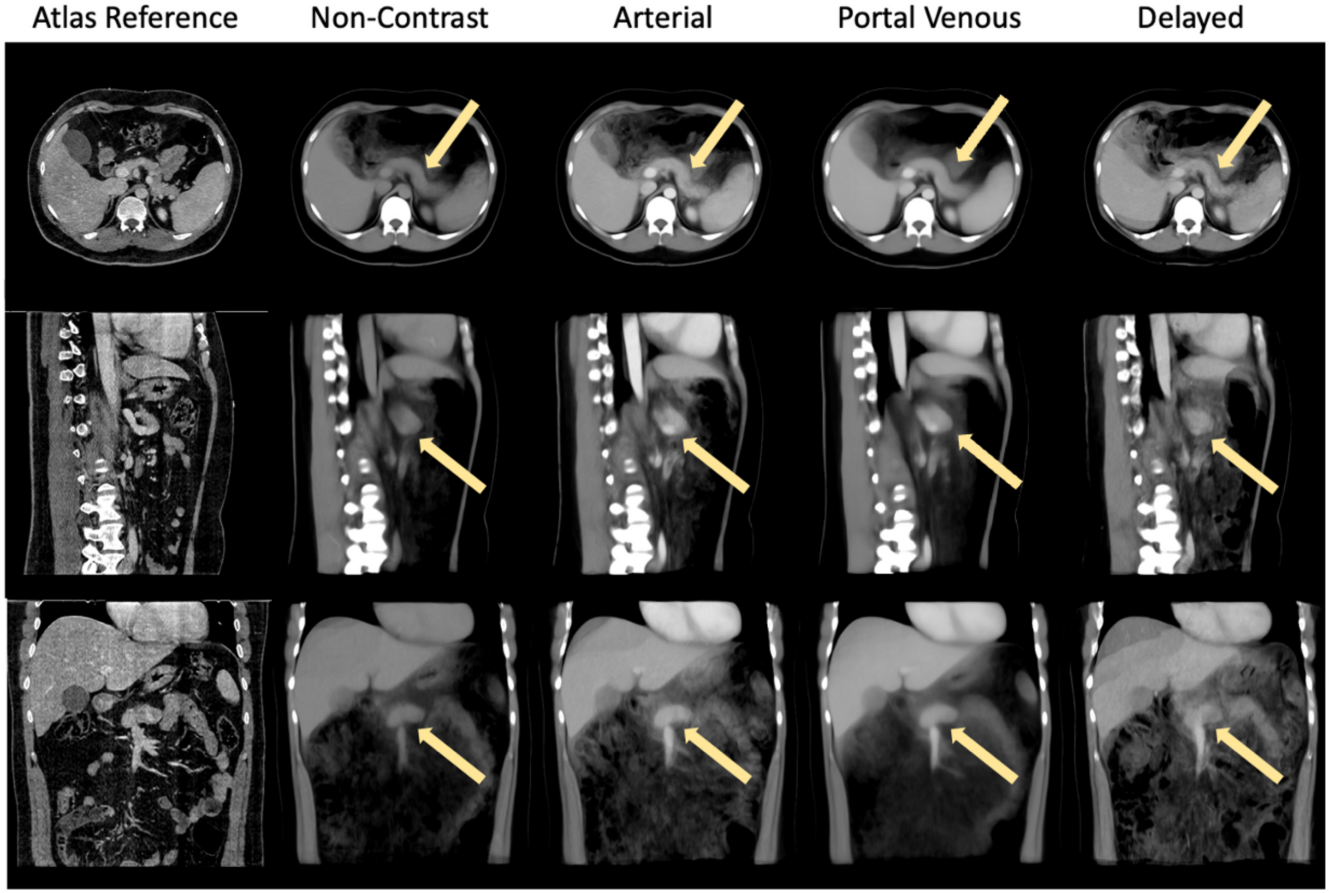
Constructed atlases for the four contrast phases are shown. The yellow arrow points to the pancreas region. We observed that the morphology of the pancreas across all phases is well preserved with clear boundaries.

**Fig. 5:**
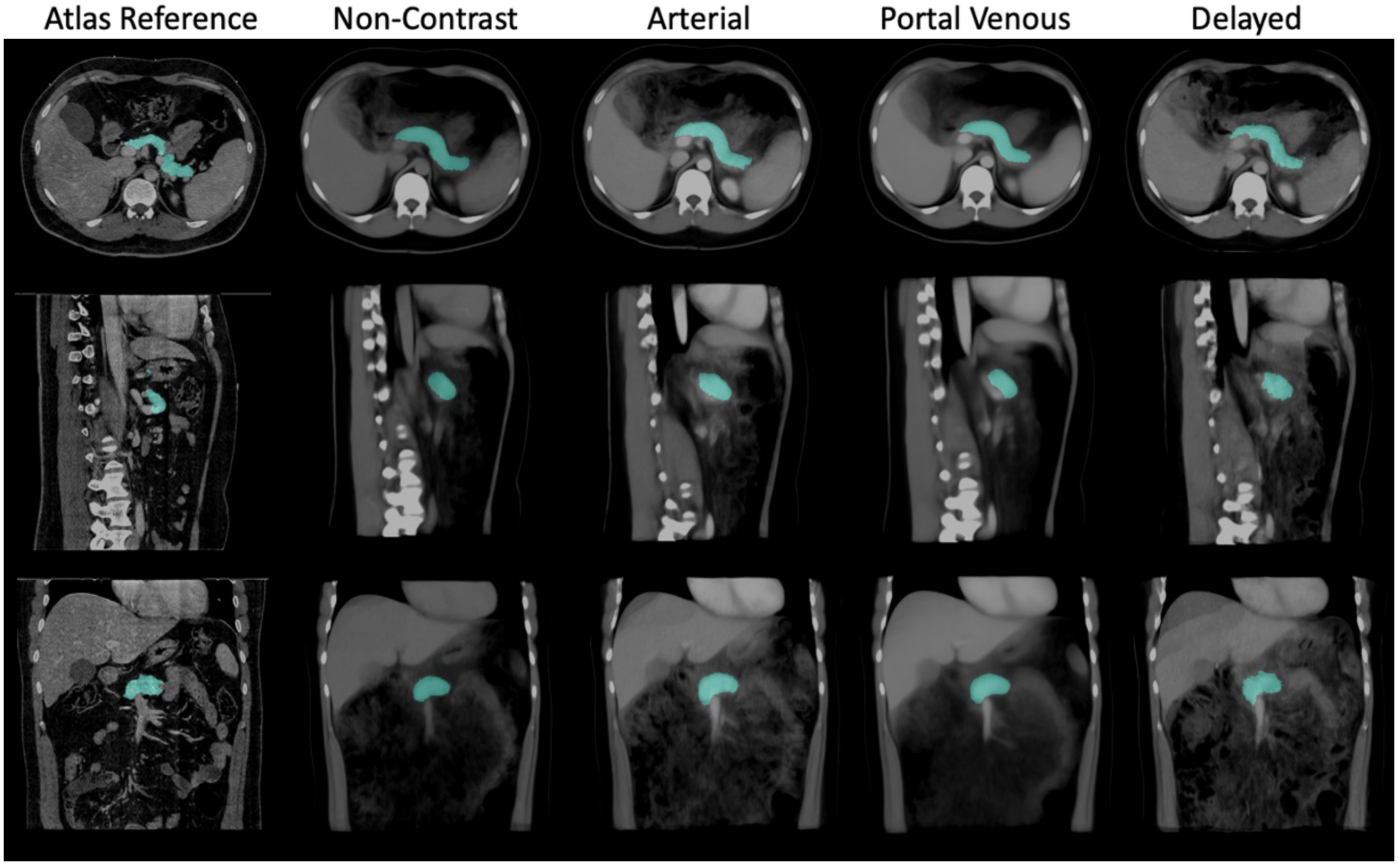
Fused pancreas label is overlaid on the constructed atlases to better localize and visualize the pancreas region across all contrast phases. The morphology of the pancreas is maintained throughout the different contrast phases.

**Fig. 6:**
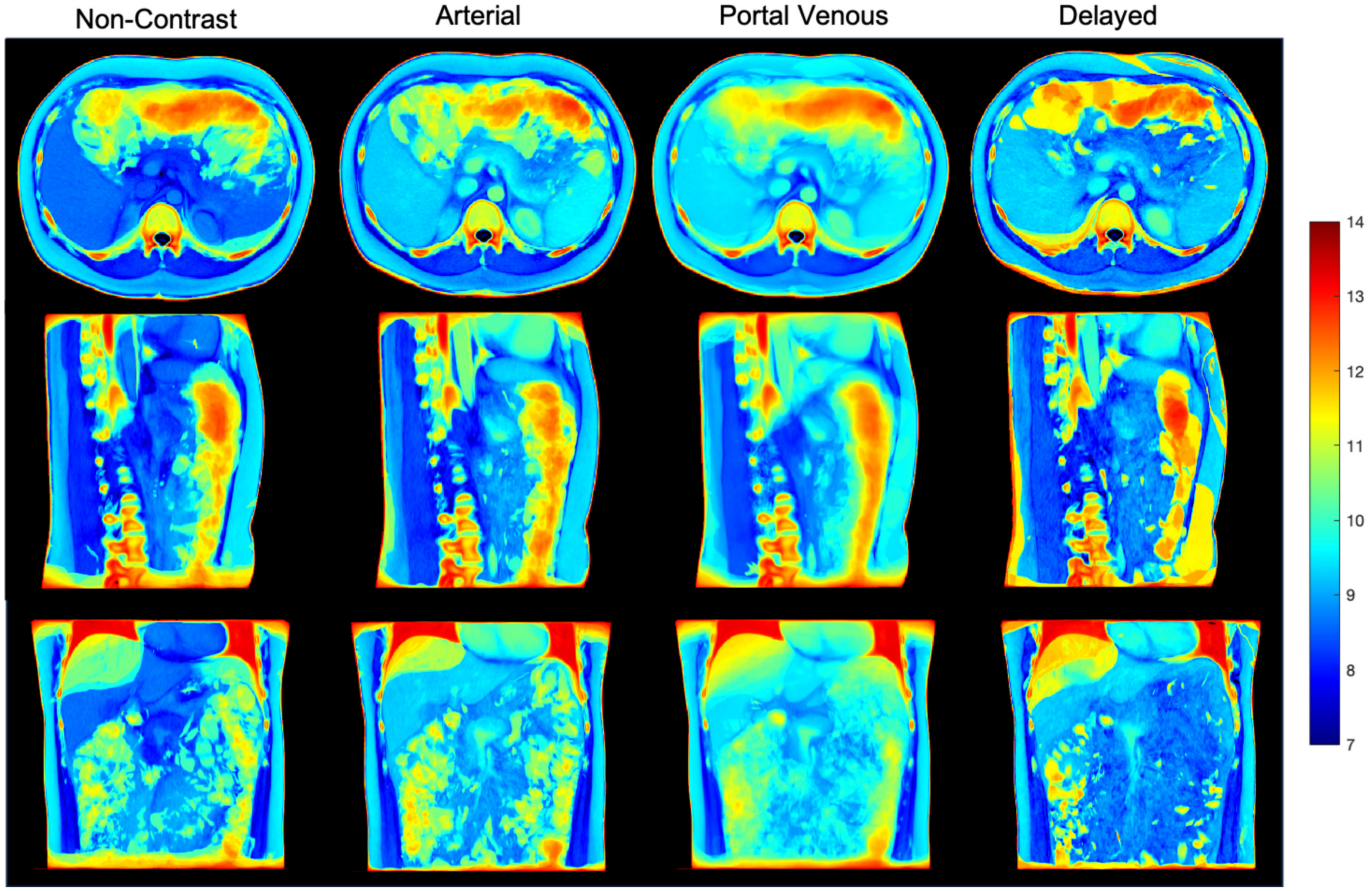
We further evaluated the intensity variance across the registration outputs and the average template in each contrast phase. The variance value outside the body was set to zero. The variance mapping in all four phases demonstrates the pancreas context transferal with stability and low variance value.

Figures 4 and 5 show a tri-planar view of the average atlas, in which the anatomical features of the pancreas can be clearly seen in all directions. Furthermore, the shape of surrounding organs, such as the liver, is well-preserved, providing a better anatomical context for studying the pancreas. In particular, the average atlas in the non-contrast and portal venous phases provides a clear view of all three planes. However, the delayed phase near the pancreas region appears slightly blurry in the coronal plane, likely due to the smaller number of subjects used to create the atlas, where only 14 volumes were used. We also calculated the variance between the average template and the registered subjects used to create the atlas. The variance of the average templates is shown in Fig. 6. Among all registered regions, the pancreas exhibits relatively low variance for all phases compared to other anatomical structures, such as the spleen and spine. To enhance the identification and visualization of the pancreas region in the atlases, segmentation labels were acquired by employing a label fusion technique that relies on majority voting. As depicted in Fig. 5, the position and shape of the pancreas in the multi-phase atlases closely resemble the target reference, indicating that the anatomical and contextual information of the pancreas has been effectively transferred to the target space. Even the areas of greatest variation, such as the head and tail of the pancreas, are distinctly visible in the fused labels. Minor differences can be observed between the segmentation labels on the atlases across the four phases, suggesting that the anatomical features and contrast properties have been maintained in the multi-phase atlases.

The quality of the obtained atlas using the DEEDS registration method for different phases was also evaluated with inverse label transformation. The pancreas segmentation labels from the atlas were inversely transformed back to the subject space, and the inverse labels were compared with the segmentation labels in the subject space. Table 1 displays the performance of various registration tools (ANTS, NIFTYREG, and DEEDS) on the subjects that were used to create the average atlas for the four phases. Note that the evaluation metrics were only calculated on the selected subjects that have a Dice score above 0.1 for all three two-stage registration methods to ensure the quality of the constructed average atlases. The two-stage DEEDS with affine and deformable registration outperformed other registration methods in terms of Dice score and Hausdorff Distance (HD), achieving an average Dice score of 0.5 and an average HD of 36.63 mm across all contrast phases in the pancreas. By contrast, other registration methods only achieved an average Dice score below 0.35 and an average HD above 43 mm, indicating the effectiveness of DEEDS. This evaluation also demonstrates high registration accuracy in the pancreas region, thereby confirming the reliability of our constructed atlas and the fused pancreas label overlaid on the atlas.

**Table 1:** Inverse label transfer performance of different registration methods on the pancreas for subjects selected to create the average atlas after quality assessment. It includes 443 subjects in total for four contrast phases from the clinical research multi-phase CT cohort. The pancreas labels are obtained from the UNesT segmentation model.

	Non-contrast		Arterial		Portal venous		Delayed	
Methods	Dice ↑	HD (mm) ↓	Dice ↑	HD (mm) ↓	Dice ↑	HD (mm) ↓	Dice ↑	HD (mm) ↓
ANTS (A)	0.256±0.115	47.9±22.1	0.242±0.103	39.9±16.8	0.242±0.082	42.2±18.0	0.249±0.078	42.5±20.1
NIFTYREG (A)	0.232±0.141	52.8±22.8	0.200±0.131	47.8±17.3	0.201±0.117	46.4±19.0	0.211±0.103	47.5±15.9
DEEDS (A)	0.192±0.103	48.7±17.8	0.144±0.112	46.4±12.6	0.148±0.111	45.3±14.5	0.150±0.094	49.0±16.5
ANTS (A+D)	0.288±0.121	47.2±22.4	0.302±0.114	38.8±17.1	0.308±0.097	40.1±18.4	0.318±0.065	41.3±20.1
NIFTYREG (A+D)	0.314±0.154	51.7±22.2	0.334±0.180	45.7±16.3	0.350±0.176	45.6±19.1	0.378±0.150	46.8±17.8
DEEDS (A+D)	0.497±0.076 ^a^	39.2±21.9 ^a^	0.505±0.075 ^a^	32.5±14.7 ^a^	0.494±0.077 ^a^	33.3±17.5 ^a^	0.497±0.086 ^a^	37.1±20.7 ^a^

a p<0.0001, Wilcoxon signed-rank test, A: affine registration, D: deformable registration.

**Fig. 7:**
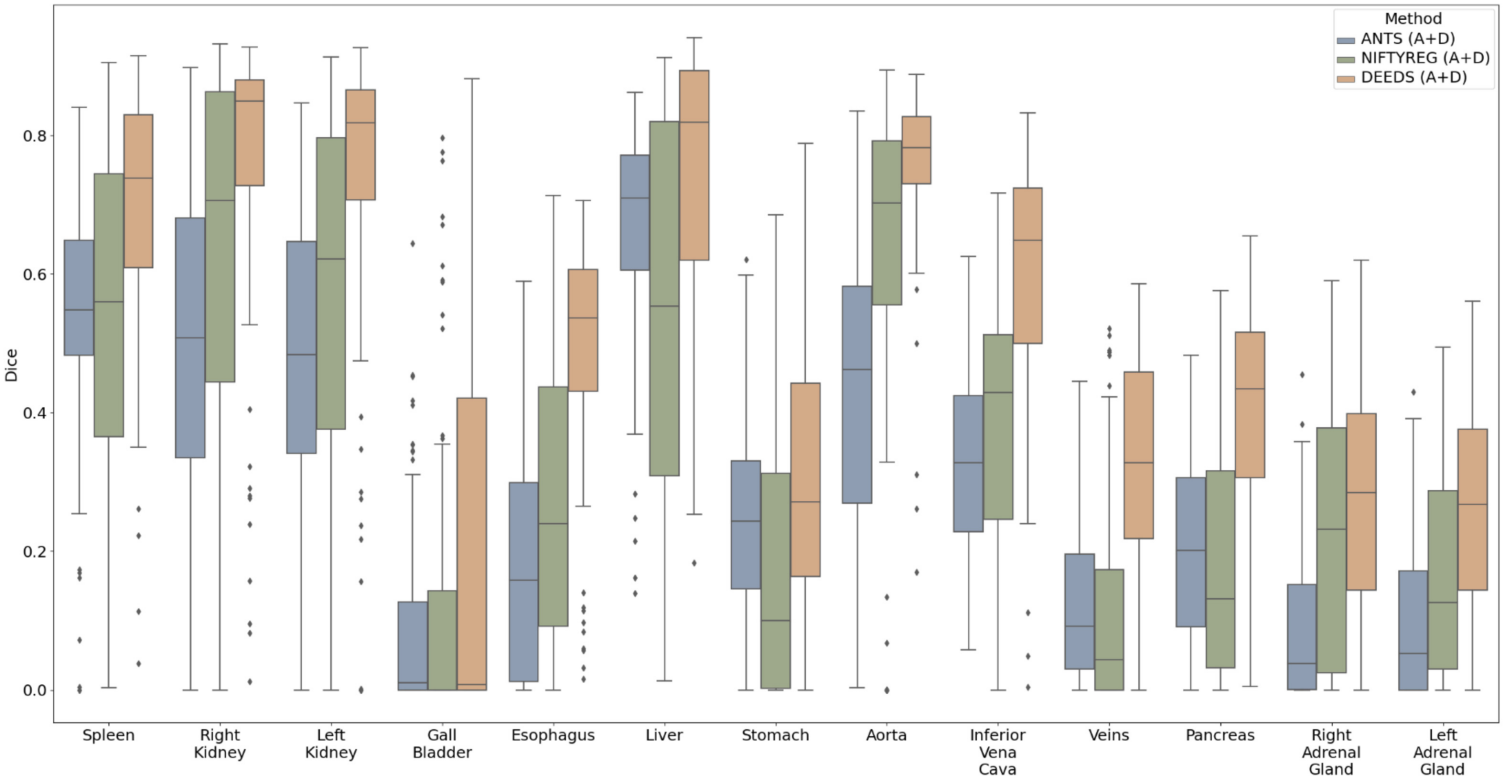
Three registration methods were evaluated on 100 subjects from the labeled multi-organ portal venous BTCV dataset. The Dice scores of the inverse label transfer indicate that DEEDS performs better than ANTS and NIFTYREG on all organs.

However, the registration performance was evaluated on the selected subjects with a quality assessment for multi-phase CT data. To fairly evaluate the performance of different registration tools, we used all 100 subjects from the multi-organ labeled BTCV data in the portal venous phase without removing any subjects exhibiting registration failure. Unlike the multi-phase CT data, which only has pseudo-labels, this dataset includes ground truth labels for 13 abdominal organs. The Dice scores for 13 abdominal organs after inverse label transformation were calculated and are displayed in Table 2 and Fig. 7. With the two-stage registration, DEEDS achieved significantly higher performance than ANTS and NIFTYREG for all 13 organs, with a mean Dice score of 0.504. For the pancreas, two-stage DEEDS achieved an average Dice score of 0.40, whereas two-stage ANTS and two-stage NIFTYREG only achieved average Dice scores of 0.20 and 0.18, respectively.

**Table 2:** Dice scores of 13 inversely transformed organ labels with different registration methods on 100 subjects from the multi-organ labeled portal venous phase BTCV data.

Organs	ANTS (A+D)	NIFTYREG (A+D)	DEEDS (A+D)
Spleen	0.536±0.175	0.544±0.241	0.697±0.178 ^a^
Right kidney	0.498±0.226	0.611±0.290	0.756±0.207 ^a^
Left kidney	0.484±0.206	0.556±0.287	0.729±0.224 ^a^
Gall bladder	0.091±0.139	0.113±0.206	0.211±0.283 ^a^
Esophagus	0.183±0.167	0.270±0.201	0.491±0.166 ^a^
Liver	0.662±0.158	0.538±0.287	0.746±0.180 ^a^
Stomach	0.256±0.138	0.175±0.198	0.315±0.208 ^a^
Aorta	0.431±0.201	0.615±0.264	0.756±0.200 ^a^
Inferior vena cava	0.323±0.143	0.379±0.196	0.596±0.173 ^a^
Veins	0.121±0.110	0.111±0.143	0.329±0.165 ^a^
Pancreas	0.204±0.132	0.184±0.170	0.398±0.168 ^a^
Right adrenal gland	0.092±0.110	0.222±0.182	0.274±0.169 ^a^
Left adrenal gland	0.095±0.111	0.162±0.151	0.253±0.155 ^a^
Average	0.306±0.246	0.344±0.293	0.504±0.280 ^a^

a p<0.0001, Wilcoxon signed-rank test, A: affine registration, D: deformable registration.

### 4.2 Ablation Study

**Fig. 8:**
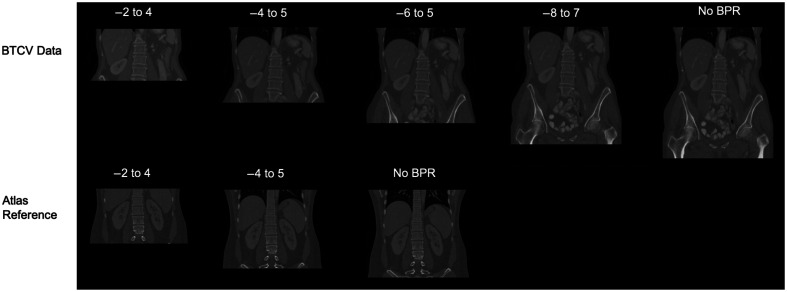
Visualization shows an example of the cropped multi-organ BTCV data and the reference subject with different BPR volumes. The cropped scans with a BPR value of −6 to 5 already have a similar field of view to the reference subject without BPR.

In our ablation study, we further investigated the influence of BPR on the performance of DEEDS registration with different ranges of cropping values. We evaluated five distinct cropping ranges to establish a similar field of interest between the moving subjects and the reference subject. The raw data without BPR provides a comprehensive view of the pelvis, abdomen, and chest regions. The cropping value of −8 to 7 encompasses most of the pelvic area and the majority of the chest area. The cropping value of −6 to 5 excludes the pelvic region and some of the chest region. The cropping value of −4 to 5 retains solely the abdominal region, whereas the cropping value of −2 to 4 effectively preserves the pancreas and kidney regions but does not entirely maintain the liver region. As shown in Fig. 8, the cropped scans with the BPR values of −6 to 5 already have a similar field of view (FOV) to the reference subject without cropping. Therefore, in the experiments without BPR and with the BPR values of −6 to +5 and −8 to +7, we cropped only the moving subjects and used the original reference volume as the target image. For the experiments involving cropping values of −4 to +5 and −2 to +4, we cropped both the reference volume and the moving volume.

**Fig. 9:**
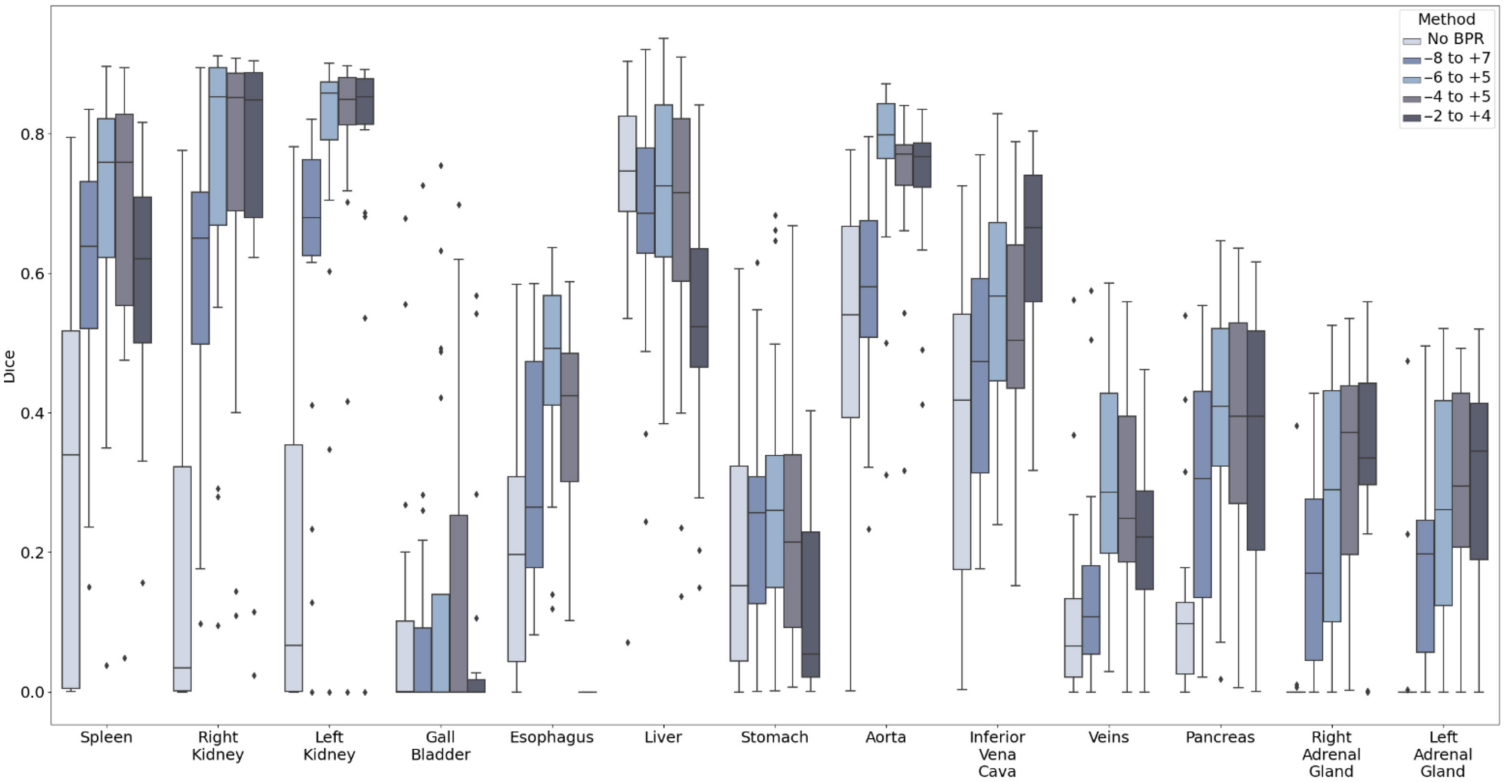
We performed an ablation study to evaluate inverse label transfer performance with different cropping value ranges for body part regression on 20 subjects from the labeled multi-organ BTCV data. We found that the optimal BPR range resulting in the best registration performance for two-stage DEEDS is between −6 and +5.

The ablation study is performed on 20 testing subjects from the labeled BTCV data, and Fig. 9 displays the inverse Dice scores for 13 labeled abdominal organs. These 20 testing subjects were cropped with different BPR values and then registered to the cropped or original target image with the same two-stage DEEDS registration process. The cropping value of −6 to +5 resulted in the highest average Dice score on the 13 organs and the highest value for the pancreas. Consequently, we employed the −6 to +5 value to crop multi-phase subjects and computed the average atlas from them. In addition, the value of −6 to +5 maintains a complete view of all abdominal regions, facilitating a more comprehensive analysis of abdominal organs within an anatomical context.

## 5 Discussion

Population-based tissue maps play a crucial role in studying human organs by generalizing the variability between individuals. In abdominal scans, the heterogeneity of abdominal and retroperitoneal scans presents challenges in constructing an atlas capable of visualizing anatomical features and spatial relationships between organs. This study aims to propose a framework for constructing an abdominal atlas optimized for the pancreas region in multi-contrast CT.

Given the varying anatomical features across different phases of multi-contrast CT, it is essential to generalize the characteristics for each phase rather than using a single atlas template. The non-contrast phase involves acquiring images before injecting the contrast agent and serves as the baseline reference. For the pancreas, the arterial and portal venous phases are frequently used for detecting and characterizing lesions when blood vessels and abdominal organs have been further enhanced by the contrast agent.^43^^,^^44^

To transfer the anatomical characteristics of each phase, we registered individual subjects in different phases to the high-resolution atlas template. The robustness of registration tools is vital for the quality of the average atlas. A two-stage registration was used, where the affine registration provides prior information for the deformable registration in the second stage. As shown in Table 2, DEEDS registration achieved an average Dice score of 0.504 for 13 organs across 100 subjects from the BTCV dataset, demonstrating accurate transfer of anatomical information for all abdominal organs. ANTs are the least robust, potentially due to their surface-based registration nature. When using ANTs for label registrations, their performance improves, but 3D intensity images are still problematic due to partial matching, especially in aligning the boundaries of abdominal organs. NIFTYREG slightly outperformed ANTs, employing a block-matching approach for non-linear registration, which provides more accurate results in the presence of large deformations but requires longer computation time. DEEDS, a voxel-based method, surpassed the other two methods in performance, possibly because it relies on discrete optimization, allowing greater control over the displacement space.^40^ One advantage of DEEDS is its use of dense stochastic sampling approaches, sampling random voxels from non-overlapping cubes, and then calculating the displacement on cubes. It ensures accurate registration of small anatomical features undergoing large motion. In addition, discrete optimization reduces computational complexity, making it more efficient than continuous optimization.

Nonetheless, registration accuracy is still less than ideal and could influence the quality of the average atlas. There were many registration failure cases for multi-phase CT scans. Some of them were completely not aligned or the pancreas region was mismatched. After the visual assessment and removing cases with very low Dice scores in the pancreas region, it reduced the usable subjects to make the atlas from 898 subjects down to 443 subjects. Even using the registration tool DEEDS with the highest performance, Table 1 shows the inverse dice of the pancreas on selected 443 subjects is still around 0.5, which still needs further improvement. Challenges for these registration tools include (1) the dissimilar field of view between moving subjects and the target template and (2) the variation of secondary structures (e.g., muscles, bones) across the population. Failure cases happen in thin people and scans with artifacts. Many registration failures also occur due to mismatched fields of view between subjects, necessitating better pre-processing steps. In the ablation study, we demonstrated the effectiveness of body part regression in matching the field of view. Secondary structures could also distract from the registration of target organs. The variation of these parts could cause undesirable deformation, especially when they occupy a large space in abdominal scans.^25^ Similarly, small or medium-sized organs could be affected by surrounding organs. For instance, pancreas deformation might be negatively affected by other surrounding target organs with larger sizes, such as the liver. By excluding registration outliers and selecting subjects with good performance in the abdominal regions, particularly the pancreas, we are able to create high-quality atlases for each contrast phase. However, exploring more robust registration tools could potentially allow the inclusion of more subjects, leading to even better average atlases.

In addition to traditional optimization-based registration, learning-based registration methods have gained popularity due to their fast speed. However, concerns remain regarding the use of learning-based methods for constructing average maps in our study. Optimization-based models generally exhibit better expressive power because model parameters are optimized for a specific pair of images, enabling sharper deformation to preserve the details of anatomical features of abdominal organs. On the other hand, learning-based methods optimize the model for the entire dataset, which tends to produce over-smoothed transformations, potentially losing individual characteristics during registration and making them less suitable for high-resolution images.^29^ Furthermore, learning-based methods typically exhibit less generalizability and are more specific to the dataset they are trained on.

In addition to constructing average atlases, fused labels on the atlas provide clearer anatomical characteristics and contextualization of the pancreas. As demonstrated in Fig. 6, the shape and position of average segmentation labels closely resemble those in the atlas template. However, limitations still exist. As the segmentation represents the average of subjects, it is not specific to individual subjects; thus, detailed features and irregular boundaries may not be accurately represented on the fused labels, providing only an estimated shape of the pancreas. Another concern regarding segmentation labels on atlases is the accuracy of the segmentation performance of the model. We employed UNesT, which was trained on the BTCV dataset in the portal venous phase. However, due to minor differences in anatomical features between subjects in each phase, segmentation in other phases may not be as accurate as in the portal venous phase.

## 6 Conclusion

This study introduces a high-resolution pancreas atlas framework to generalize healthy biomarkers across populations with multi-contrast abdominal CT. By utilizing the deep neural network BPR to match the field of view between the target template and moving subjects and employing the DEEDS registration method to transfer subjects into the target space, our atlas effectively captures population-wide features of the pancreas organ and contextualizes the anatomical characteristics of the pancreas within the entire abdominal scans. Future work involving the use of pancreas atlases could further explore areas such as enhancing the segmentation accuracy of the pancreas and improving the localization of the pancreas in the context of pathological changes.

## Data Availability

The data used for this article, including multi-phase contrast CT images, are not publicly available. The average template and the associated pancreas organ labels are publicly available through the Human Biomolecular Atlas Program (HuBMAP).
